# Reward History and Multisensory Signals Interact in a Localisation Task

**DOI:** 10.1177/17470218261452613

**Published:** 2026-05-11

**Authors:** Bryan Sim, Ada Kritikos, Mick Zeljko

**Affiliations:** 1 School of Psychology, The University of Queensland, St. Lucia, Australia

**Keywords:** multisensory integration, reward history, value-modulated attentional capture, response selection, localisation

## Abstract

Previously rewarded stimuli can influence later performance even when they are task-irrelevant, while multisensory stimuli often enhance perceptual salience and speed responses to targets. This study examined how reward history and multisensory stimulus configuration jointly shape behaviour in a speeded localisation task. Participants first learned associations between visual stimuli and high or low reward. In a subsequent test phase, they localised an unrewarded visual target while a previously rewarded distractor appeared in the opposite hemifield. Target and distractor modalities were independently varied between unisensory visual and multisensory audiovisual stimuli. Responses were faster to multisensory than unisensory targets, and high-value distractors slowed responses relative to low-value distractors when both target and distractor were unisensory. However, when targets were multisensory and distractors were unisensory, distractor value no longer influenced response times. Multisensory signals at the distractor produced more complex effects: when the target was multisensory, high-value multisensory distractors slowed responses more than high-value unisensory distractors, whereas when the target was unisensory, high-value multisensory distractors produced less slowing than high-value unisensory distractors. These findings show that reward history and multisensory stimulus configuration interact in shaping target-localisation performance. The observed effects may reflect attentional selection, response selection, or interactions between these processes, but not necessarily attentional selection. To determine this, future designs should disambiguate target location and response.

## Introduction

In complex, ever-changing sensory environments, behaviour is shaped by both multisensory integration (MSI) and mechanisms that prioritise relevant information over competing input. Through MSI, inputs from different sensory modalities are combined to form coherent and robust perceptual representations (Frassinetti et al., 2002; Gingras et al., 2009; Rowland & Stein, 2008; Zeljko & Grove, 2021, Zeljko et al., 2021), manifesting in reliable behavioural benefits. For example, in the redundant targets effect (Miller, 1982), responses are faster to concurrent (bimodal) auditory and visual cues than to the same cues presented unimodally (Giard & Peronnet, 1999; Molholm et al., 2002; Stein & Meredith, 1993). In parallel, selective-attention mechanisms bias processing toward task-relevant stimuli and away from competing input (Desimone & Duncan, 1995; Driver, 2001; James, 1890; Treisman & Gelade, 1980). This is achieved through endogenous, top-down, mechanisms involving conscious allocation of mental resources (Awh et al., 2012; Corbetta & Shulman, 2002; Posner, 1980), as well as exogenous, bottom-up mechanisms involving the involuntary prioritisation of salient or novel stimuli (Theeuwes, 2010; Yantis & Jonides, 1984).

MSI also interacts with both stimulus-driven and goal-directed processing (Talsma et al., 2010; Tang et al., 2016). First, voluntary spatial attention enhances MSI, such that audiovisual stimuli appearing in an endogenously cued location evoke larger ERP responses than identical stimuli in unattended space (Talsma & Woldorff, 2005). Moreover, Mozolic et al. (2008) observed robust audiovisual interactions only when participants divided attention across both modalities. Second, multisensory signals can facilitate visual search and orienting. In the “pip-and-pop” effect, a spatially uninformative sound presented simultaneously with a difficult-to-find visual target accelerates visual search (van der Burg et al., 2008). Similarly, Santangelo and Spence (2007) showed that audiovisual cues captured attention under high perceptual load, whereas visual-only and auditory-only cues did not. They proposed that perceptual load raises the saliency threshold for exogenous orienting, and multisensory signals exceed that threshold. By boosting perceptual sensitivity and target saliency, MSI may help resolve competition among stimuli and support responses to behaviourally relevant events (Tang et al., 2016).

Directly relevant to this study, interactions between sensory processing (unisensory and multisensory), stimulus selection, and response performance can be influenced by low-level salience and immediate goals (Awh et al., 2012; Corbetta & Shulman, 2002; Desimone & Duncan, 1995; Talsma et al., 2010), and by higher-level processes such as learning and memory (Anderson et al., 2011a; Gilbert & Li, 2013; Shams & Seitz, 2008; Summerfield & Egner, 2009; Ten Oever et al., 2016). In particular, reward learning can influence multisensory processing, stimulus prioritisation, and response performance in different ways. For example, associating rewards with stimuli to be integrated can either impair (Bruns et al., 2014; Sanz et al., 2018) or enhance (Bean et al., 2021) MSI depending on the task. Reward-associated stimuli have been shown to influence later target selection and performance in attentional-capture paradigms long after rewards cease (Anderson & Yantis, 2013; Failing & Theeuwes, 2017; Sanz et al., 2018). This phenomenon, known as value-driven or value-modulated attentional capture (VDAC/VMAC), occurs even if these previously rewarded stimuli are not relevant to the current task, are not physically more salient, and offer no current incentive. Here, we explore how sensory processing and reward history, exemplified by MSI and VMAC-related paradigms, may interact to shape target-localisation performance.

### Reward Learning and VMAC

Responses to reward-associated stimuli can be faster and more accurate than responses to non-rewarded stimuli (Kiss et al., 2009), and reward cues can bias processing by enhancing relevant objects and suppressing distractors (Libera & Chelazzi, 2009). Reward has been proposed to guide selection by modifying motivation and voluntary control (Pessoa & Engelmann, 2010; Serences, 2008). It may also increase the priority of reward-associated stimuli (Anderson & Yantis, 2013; Hickey et al., 2010), thereby engaging exogenous attentional mechanisms. Further, even when reward ceases to be task-relevant, previously rewarded stimuli can continue to influence later target selection and response performance. For example, a previously high-value target that is now a distractor can slow selection of a new target in subsequent trials, termed VDAC (Anderson et al., 2011a, 2011b). Anderson et al. (2011a) had participants initially undergo a training phase in which they made rapid searches for a red or green target amongst other coloured distractors. After each trial, they received visual feedback indicating a monetary reward for a correct response where high reward was associated with one colour and low with the other. During the subsequent testing phase, participants made rapid searches for a uniquely shaped target amongst other shaped distractors. Importantly, on half of the trials, one of the distractors was red or green. Participants were told to ignore colour and no reward was provided. Previously rewarded stimuli were now distractors, and selection of, or responding toward, these distractors would hinder performance on the target task. The presence of the previously rewarded distractors consistently slowed target detection compared with a neutral but equally salient colour distractor. Further, high-reward distractors impaired reaction times to a greater extent than low-reward distractors. Le Pelley et al. (2015) later demonstrated that the high-value distractors remain effective not only when task-irrelevant, but even when ignoring them would maximise reward in the current task.

Awh et al. (2012) suggested that VMAC is neither purely top-down nor bottom-up (see also Failing & Theeuwes, 2017, 2018). Rather, it has been proposed to emerge from selection history, creating a lasting bias toward previously rewarded features. They propose that reward is one element of a broad set of history effects, including priming, statistical learning, and emotional conditioning, that bias attentional priority maps. Thus, selection may be influenced not only by current goals and stimulus salience, but also by prior selection history.

### Multisensory Integration and Reward Learning

Reward also influences MSI. Sounds previously paired with high compared with low monetary reward improved the orientation discrimination of a concurrent visual stimulus, suggesting persistent effects of reward associations on MSI (Pooresmaeili et al., 2014). In a study on cats, Bean et al. (2021) found that MSI and its associated stimulus detection benefits emerged only if the stimulus components were novel or had been previously rewarded. If one stimulus component was consistently unrewarded, multisensory benefits were abolished, even with spatiotemporally congruent stimuli.

The ventriloquism effect, where a simultaneous but spatially offset auditory stimulus is perceived as spatially closer to a visual stimulus than it is, was initially viewed as automatic and bottom-up (Bertelson et al., 2000; Vroomen et al., 2001). More recently, reward learning has been shown to moderate this effect.^1^ Bruns et al. (2014) had participants locate an auditory stimulus presented in either the left or right hemifield, where each hemifield was associated with a high or low reward. The auditory stimulus was accompanied by a visual stimulus that was task-irrelevant and spatially misaligned. Participants were rewarded for correct localisations, and high-low reward difference moderated the strength of the ventriloquism effect, with MSI weaker for stimuli in the high- compared with the low-reward hemifield. Bruns et al. (2014) suggested that reward learning influences the probability that two stimuli are perceived to originate from a single source and biases processing toward the rewarded modality (auditory) and hemifield.

Relevant to this study, Sanz et al. (2018) investigated the role of reward in MSI and crossmodal effects on VDAC. In an initial association phase, two different sounds (a trumpet and a drum) were paired with high and low monetary rewards through a speeded localisation task that rewarded correct responses. In the subsequent testing phase, participants made speeded localisation discriminations of an image of either a trumpet or drum presented either to the left or right of fixation. Critically, before image onset, one of the previously rewarded sounds was played either to the left or right ear and was semantically congruent or incongruent as well as laterally (left or right) congruent or incongruent with the forthcoming image. Response times to visual targets when stimuli were semantically congruent were significantly increased under conditions of high-reward auditory distractors. Lateral congruence did not have a significant effect on reaction times, however. Thus, high-reward distractor sounds impaired performance, which Sanz et al. (2018) interpreted as reduced MSI and attentional capture by previously rewarded auditory stimuli.

### The Current Study

While Sanz et al. (2018) found that high-reward auditory distractors can impair visual target responses in a crossmodal VDAC paradigm, there remain significant gaps in understanding how MSI and reward history interact during target localisation. Specifically, little is known about how previously rewarded information alters subsequent behaviour, and how these effects interact with multisensory stimulus configurations during target selection and response. Here, we investigate the relationship between MSI and reward history in a modified VMAC-related localisation paradigm. Participants first associate a high or low reward with two different visual stimuli during an initial phase, then they perform a speeded localisation of a novel, unrewarded visual stimulus in the presence of a previously rewarded stimulus presented in the opposite hemifield as a distractor. Critically here, either the target or the distractor, or both, could be unisensory (visual) or multisensory (audiovisual) allowing us to examine interactions between target modality (unisensory vs. multisensory), distractor modality (unisensory vs. multisensory), and distractor reward (high or low). We examine response times to correctly located targets.

First, we expected that under unisensory conditions, previously high-value distractors would prolong response times more than low-value distractors, producing value-dependent slowing consistent with the behavioural pattern typically observed in VMAC paradigms (Anderson et al., 2011b; Le Pelley et al., 2015). Separately, response times to multisensory targets should be faster than to unisensory targets (Tang et al., 2016). Second, we expected an interaction such that multisensory signals at the target would reduce value-dependent interference from unisensory distractors, consistent with evidence that multisensory cues can influence spatial orienting and search under demanding conditions (Spence & Santangelo, 2009). Third, we expected an interaction such that multisensory signals at the distractor would modulate value-dependent interference. Specifically, a multisensory high-reward distractor should increase response times to targets more than a unisensory high-reward distractor for both unisensory and multisensory targets (Bean et al., 2021). However, responses to both unisensory and multisensory targets paired with unisensory and multisensory distractors with low-value reward history were expected to be comparable; that is, we expected no response-time differences between these low-value distractor conditions. Because the required response was defined by target location, the present design cannot fully separate effects on attentional selection from effects on response selection. We therefore interpret the findings primarily as evidence about how reward history and multisensory stimulus configuration jointly shape target-localisation performance.

## Method

### Participants

Fifty first-year psychology students from the University of Queensland (33 females, 17 males, age, *M* = 19.9, *SD* = 4.0) participated in the study in return for course credit and an additional $10 (the reward manipulation). Participants had normal or corrected-to-normal vision, they each provided written informed consent prior to the experiment, and the study was approved by the University of Queensland Human Research Ethics Committee. An a priori power calculation and studies investigating MSI, reward learning, and distractor interference were consulted to determine a sample size of 50 participants was adequate in detecting a medium effect size with 80% power.

### Apparatus

All stimulus presentation and data collection were conducted using a PC running MATLAB (r2018b) with PsychToolbox (Brainard, 1997; Kleiner et al., 2007; Pelli, 1997) installed. All visual stimuli were presented on a monitor (Acer KG271, 1920 × 1080 screen resolution, 60 Hz refresh rate) and all auditory stimuli were presented via speakers that were positioned to the left and right of the monitor (48 kHz sampling rate, Logitech). Participant responses were captured using a keyboard (Corsair K68 RGB) connected to the PC. Participants were seated unrestrained approximately 60 cm from the monitor.

### Stimuli and Procedure

The experiment consisted of an association block and a testing block, each consisting of 192 trials. The association block was conducted first (Figure 1). A black fixation cross (12 pixels × 12 pixels large) was presented at the centre of the screen. After 2,000 ms, a blue (RGB: 5, 30, 200) or green (RGB: 5, 220, 255) coloured target (a circle; 4° radius; contrast and luminance matched) was presented 20° left or right of fixation for 100 ms and participants were instructed to indicate the target location as fast and accurately as possible using the left and right arrows of the keyboard. Target colour and location were evenly split between blue and green and left and right of fixation and randomly intermixed within the block. Responses were timed out after 750 ms, and the location (left or right) and response time (target onset to response) were recorded. If no response was made before the 750 ms time out, a prompt appeared as a reminder for participants to respond faster in subsequent trials. Incorrect responses triggered a prompt telling participants that they had chosen incorrectly and to respond to the target in the next trial.

**Figure 1.**
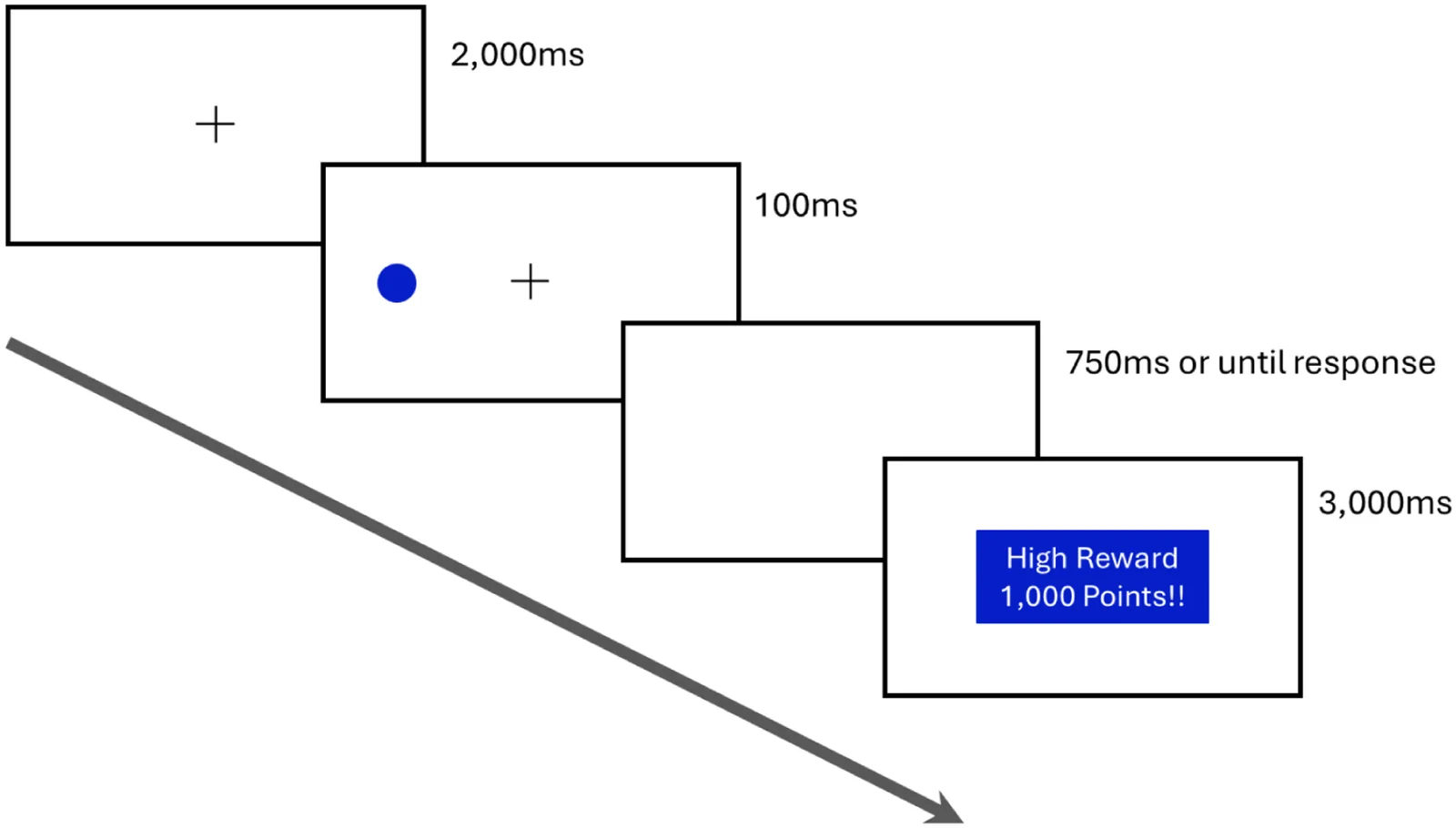
Association block illustrative trial. *Note*. Illustrative trial with a correct (left) response to the presentation of a blue target to the left of fixation with reward feedback (here, the blue target is associated with a high reward). In any single trial the target could be green or blue and presented left or right of fixation.

The reward manipulation was operationalised by target colour with one colour associated with a high reward (1000 points) and the other with a low reward (10 points), counterbalanced between participants. Participants were informed of the different reward levels, that fast and correct responses would result in points being earned, and that the points would be converted into cash at the end of the experiment. Reward feedback was provided for correct responses in the form of a message (in the same colour as the target for that trial) that informed participants of the number of points and the type of reward (high or low). To maintain participant engagement, the feedback was only provided on 80% of the correct trials; however, participants were always compensated the full $10.

The testing block was conducted after the association block (Figure 2). A black fixation cross (12 pixels × 12 pixels large) was presented at the centre of the screen. After 250 ms, a grey (RGB: 128, 128, 128) target (a circle; 4° radius) was presented 20° left or right of fixation for 100 ms and participants were instructed to indicate the target location as fast and accurately as possible using the left and right arrows of the keyboard. A distractor (a circle; 4° radius) was always presented simultaneously with the target, 20° from fixation on the opposite side to the target. The colour of the distractor was either blue or green and so was associated with either a high or low reward during the association block. Participants were informed that there were no rewards during the testing block. For some trials, a sound (1,000 Hz pure tone) could occur simultaneously with, and on the side of, the target, the distractor, or both. The testing block was a 2 (target modality: unisensory, multisensory) × 2 (distractor value: low, high) × 2 (distractor modality: unisensory, multisensory) with all trial types intermixed and each trial type presented 24 times. Participants were instructed to ignore both the distractor and the sound and to only respond to the grey target. Thus, the target location directly determined the required left/right response during the test phase.

**Figure 2.**
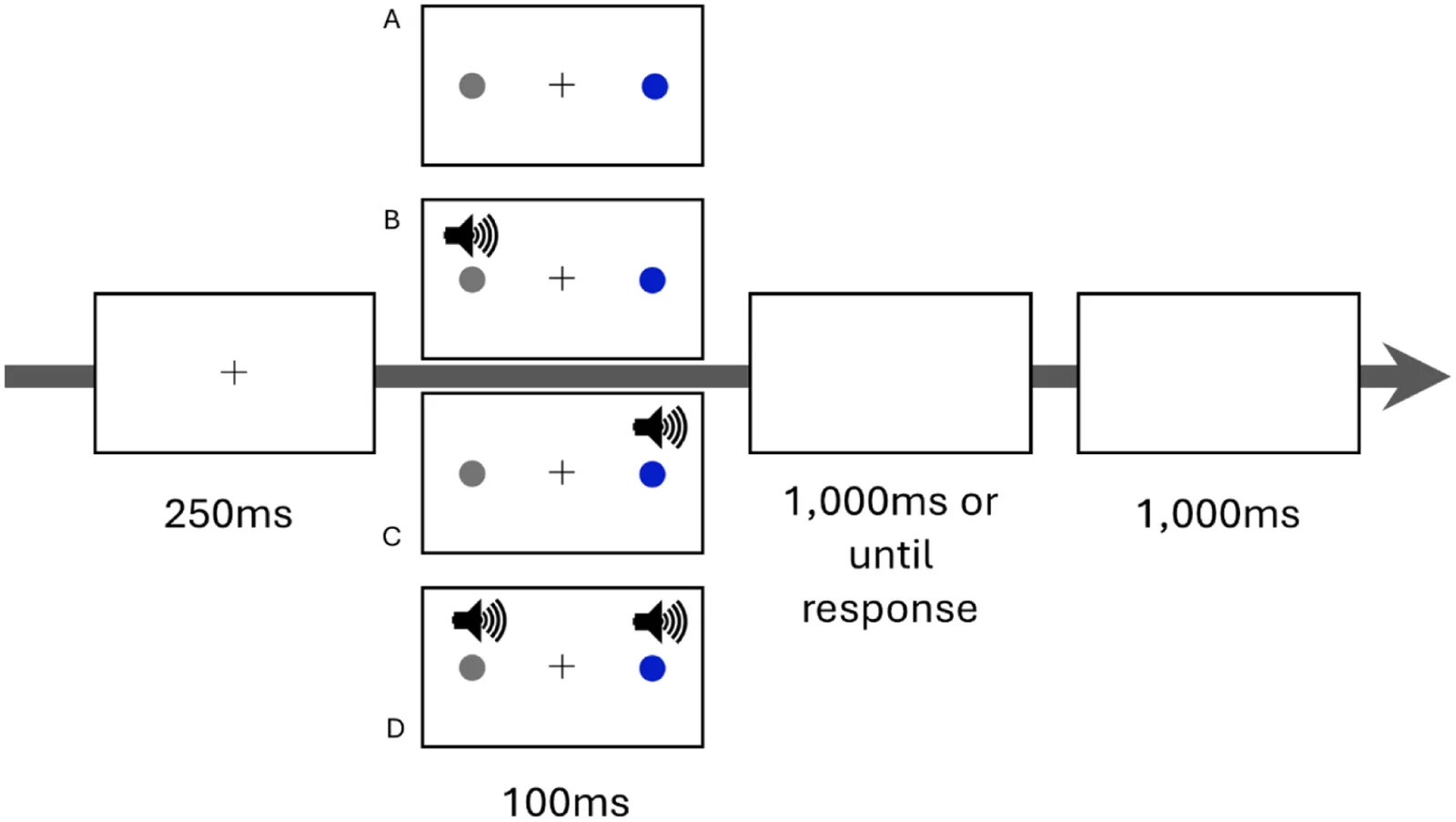
Testing block illustrative trials. Illustrative trials with the presentation of the (grey) target to the left of fixation and a blue distractor to the right of fixation: (A) the target and distractor are both unisensory, (B) the target is multisensory while the distractor is unisensory, (C) the target is unisensory while the distractor is multisensory, and (D) the target and distractor are both multisensory. In any single trial the target could be presented left or right of fixation and the distractor could be blue or green (and therefore associated with either a high or low reward depending on colour/reward assignment in the association block).

Responses were timed out after 1,000 ms, and the location (left or right) and response time (target onset to response) were recorded. If no response was made before the 1,000 ms time out, a prompt appeared as a reminder for participants to respond faster in subsequent trials. Incorrect responses triggered a prompt telling participants that they chose incorrectly and to respond to the target in the next trial. No other feedback was provided, and each trial ended with a 1,000 ms pause after the response was made or timed out.

## Results

After removing outliers (response times falling outside ±3 SDs from the global mean) and data for incorrect trials, we computed average response times for each participant for each condition and these were the basis of our statistical analyses (in total, 3.6% of trial data was removed in block 1 and 4.7% in block 2). Our analyses consist mainly of planned comparisons between conditions utilising two-tailed paired samples *t*-tests. Where there are multiple comparisons being made, *p*-values have been adjusted using the Bonferroni-Holm method.

### Association Block

As a validity check, we first examined whether reward learning occurred during the association block. We conducted a paired sample two-tailed *t*-test between the low-reward target and the high-reward target, with participants’ average response times as the dependent measure. We found a significant effect of target value with participants responding significantly faster to high (*M* = 303 ms, *SD* = 35 ms) than low-value targets (*M* = 307 ms, *SD* = 30 ms), *t*(49) = 2.27, *p* = .027, *d* = 0.32 (Figure 3).

**Figure 3.**
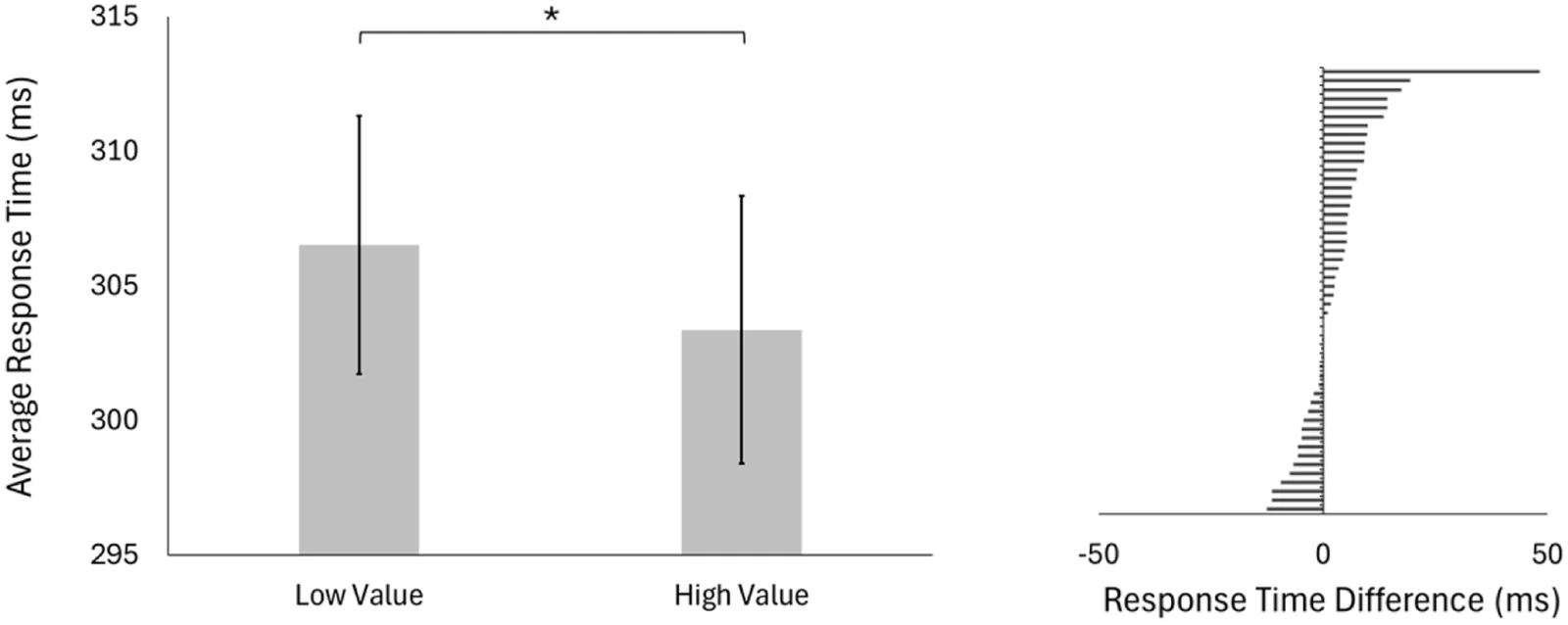
Association block: Response times to high-reward and low-reward targets. *Note*. Left: group average participant response times to low versus high-value targets during the association block (error bars are SEM – Standard Error of the Mean). Right: distribution of individual participant difference scores (average low-value RT (reaction time) – average high-value RT). *p < .05.

### Testing Block

We first conducted a 2 (target modality: unisensory, multisensory) × 2 (distractor value: low, high) × 2 (distractor modality: unisensory, multisensory) repeated measures analysis of variance (ANOVA) on the participant-averaged response time data (Table 1; Figure 4). We found a significant main effect of target modality (*F*[1, 49] = 87.51, *p* < .001; η^2^_*p*_ = .641), a significant two-way interaction between target modality and distractor modality (*F*[1,49] = 40.15, *p* < .001; η^2^_*p*_ = .450), and a significant three-way interaction between target modality, distractor value, and distractor modality (*F*[1,49] = 6.16, *p* = .017; η^2^_*p*_ = .112). All other main effects and interactions were nonsignificant (distractor value: *F*[1,49] = 0.553, *p* = .461; distractor modality: *F*(1, 49) = 1.32, *p* = .255; target modality × distractor value: *F*(1,49) = 0.286, *p* = .595; distractor value × distractor modality: *F*(1,49) = 0.012, *p* = .913).

**Table 1.** Group Average Participant Response Times (and Standard Errors) for All Conditions in the Testing Block.

Distractor modality:	Unisensory				Multisensory			
Target modality:	Unisensory		Multisensory		Unisensory		Multisensory	
Distractor value:	Low	High	Low	High	Low	High	Low	High
RT Mean (ms):	327	331	309	307	324	322	316	318
RT *SE* (ms):	5	5	6	6	6	5	6	5

**Figure 4.**
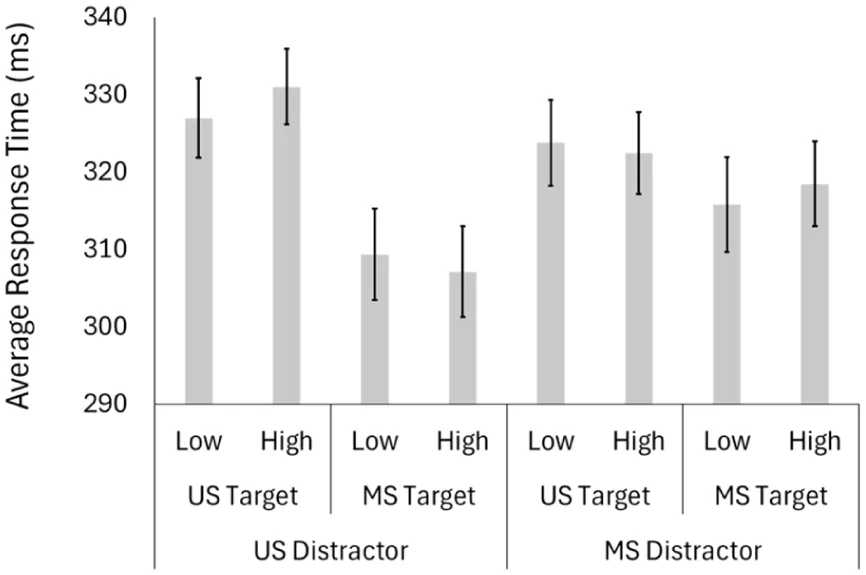
Testing block: Response times across all conditions. *Note*. Group average participant response times for all conditions in the testing block (error bars are SEM).

We first consider our replication hypotheses (Figure 5). Examining the significant main effect of target modality, we found that participants responded significantly faster to multisensory (*M* = 313ms, *SD* = 40 ms) compared with unisensory targets (*M* = 326 ms, *SD* = 36 ms), indicating a multisensory target benefit in response time. Comparing average response times to unisensory targets paired with low versus high-value unisensory targets, participants responded significantly slower to the target when the distractor was high (*M* = 331 ms, *SD* = 35 ms) compared with low value (*M* = 327 ms, *SD* = 37 ms), *t*(49) = 2.54, *p* = .043, *d* = 0.36, indicating value-dependent slowing when both target and distractor were unisensory.

**Figure 5.**
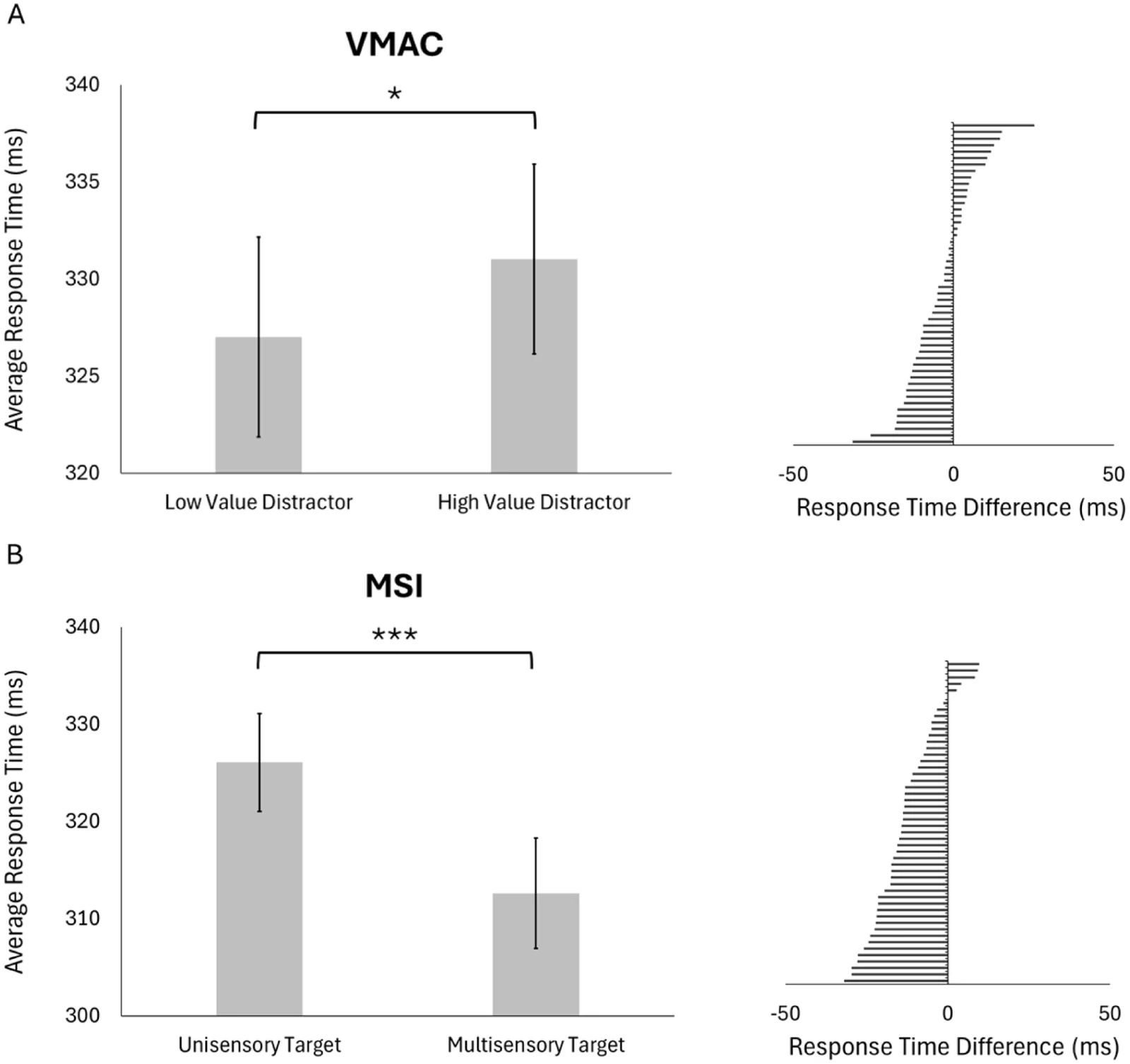
Testing block: Value-dependent slowing and multisensory target benefit: (A) Left: group average participant response times to unisensory targets paired with unisensory low-value versus high-value distractors (error bars are SEM). Right: distribution of individual participant difference scores (average low-value distractor RT – average high-value distractor RT). (B) Left: group average participant response times to unisensory versus multisensory targets during the testing block (error bars are SEM). Right: distribution of individual participant difference scores (average unisensory target RT – average multisensory target RT). *p < .05. ***p < .001.

Next, we considered whether multisensory signals at the target reduced value-dependent interference from unisensory distractors (Figure 6). Comparing average response times to multisensory targets, we found no significant difference when targets were paired with unisensory high-reward distractors (*M* = 307 ms, *SD* = 42 ms) versus unisensory low-reward distractors (*M* = 309 ms, *SD* = 42 ms, *t*[49] = 1.18, *p =* .242). When the target was multisensory, the value-dependent slowing observed under fully unisensory conditions was no longer evident. Based on the significant three-way interaction found in our main analysis, we further examine the effect of MSI at the target (with a unisensory distractor) by conducting a 2 (target modality: unisensory, multisensory) × 2 (distractor value: low, high) ANOVA. We found a significant main effect of target modality (*F*[1,49] = 107.98, *p* < .001; η^2^_*p*_ = .688), no main effect of distractor value (*F*[1,49] = 0.53, *p* = .469; η^2^_*p*_ = .011), but a significant interaction between target modality and distractor value (*F*[1,49] = 6.43, *p* = .014; η^2^_*p*_ = .116). Following up the significant interaction, we found that the multisensory benefit (the difference between response times to unisensory vs. multisensory targets) was greater when the distractor value was high compared to when it was low (*M*[high] = 24 ms, *SE*[high] = 2 ms, *M*[low] = 18 ms, *SE*[low] = 3 ms, *t*[49] = 2.54, *p* = .014, *d* = 0.36).

**Figure 6.**
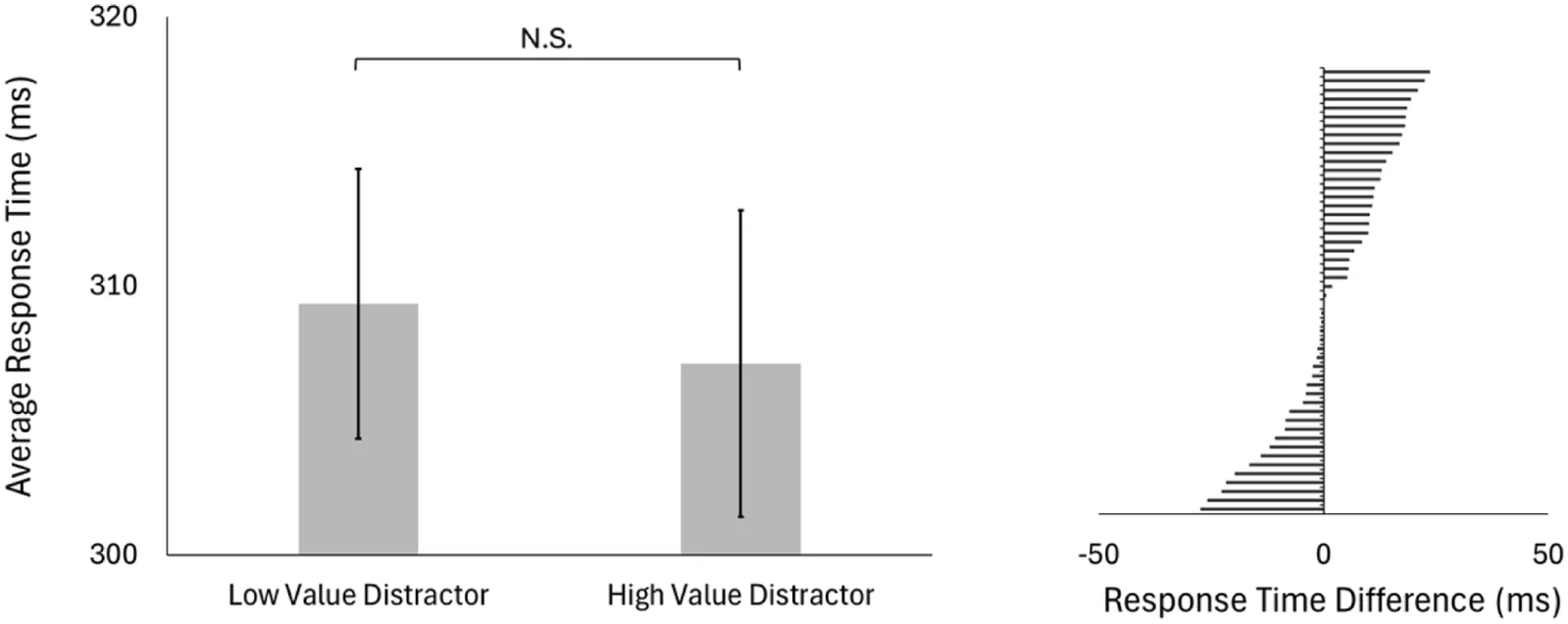
Testing block: Multisensory target signals and value-dependent interference. *Note*. Left: group average participant response times to multisensory targets paired with unisensory low-value versus high-value distractors during the testing block (error bars are SEM). Right: distribution of individual participant difference scores (average low-value distractor RT – average high-value distractor RT). NS = not significant.

Finally, we examined whether multisensory signals at the distractor modulated interference differently for high- and low-value distractors (Figure 7). We compared average response times to targets paired with unisensory versus multisensory distractors for different combinations of target modality and distractor value. For high-value distractors, responses to multisensory targets were faster when the distractor was unisensory than when it was multisensory, as predicted (unisensory distractor: *M* = 307 ms, *SD* = 42 ms; multisensory distractor: *M* = 318 ms, *SD* = 39 ms; *t*[49] = 5.15, *p* < .001, *d =* 0.73). However, the reverse pattern occurred when the target was unisensory, contrary to predictions: responses to unisensory targets were faster when the high-value distractor was multisensory than when it was unisensory (unisensory distractor: *M* = 331 ms, *SD* = 35 ms; multisensory distractor: *M* = 322 ms, *SD* = 38 ms; *t*[49] = 3.41, *p =* .007, *d =* 0.48).

**Figure 7.**
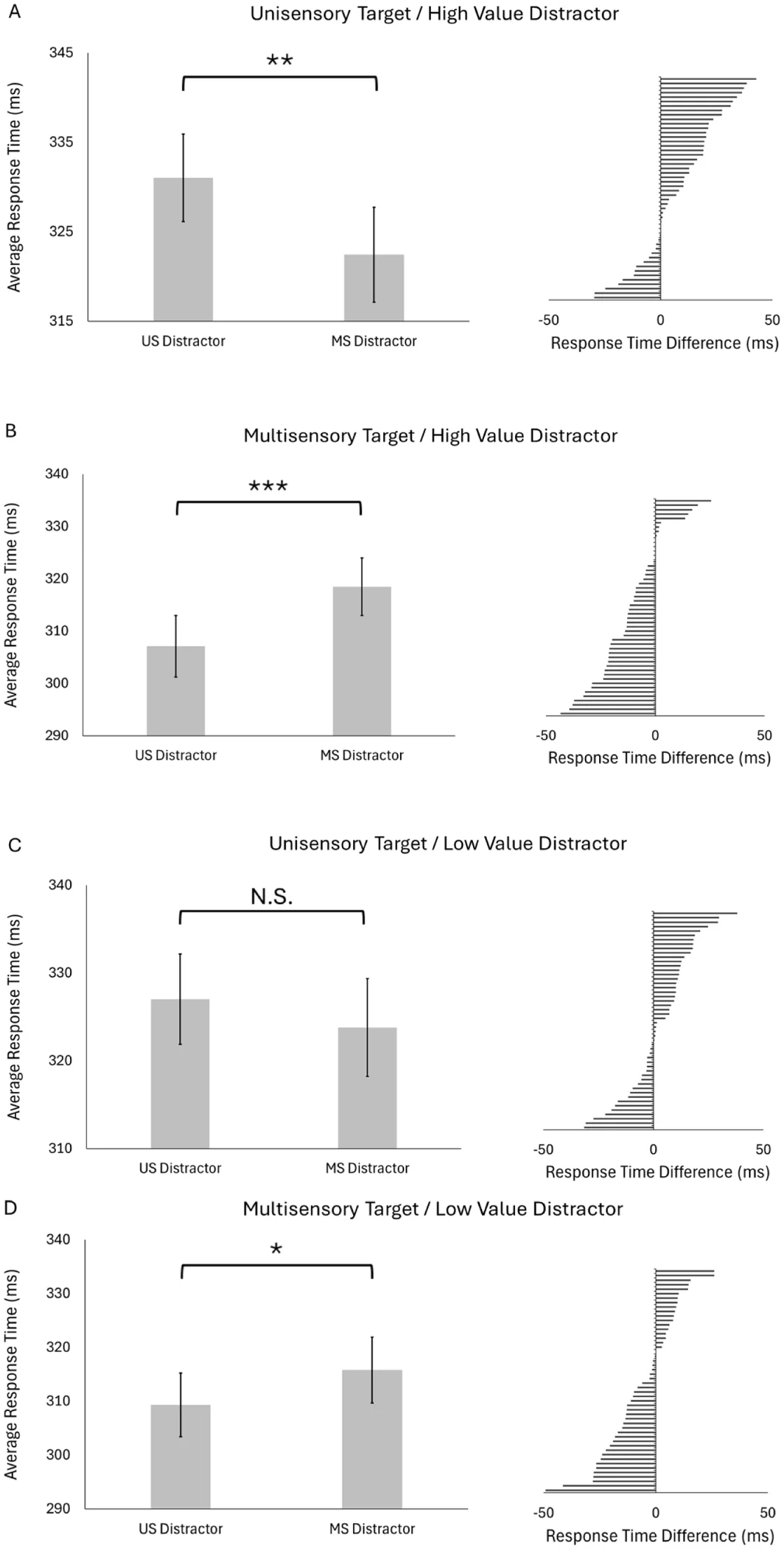
Testing block: Multisensory distractor signals and value-dependent interference. (A) Left: group average participant response times to unisensory targets paired with unisensory versus multisensory high-value distractors (error bars are SEM). Right: distribution of individual participant difference scores (average unisensory distractor RT – average multisensory distractor RT). (B)Left: group average participant response times to multisensory targets paired with unisensory versus multisensory high-value distractors (error bars are SEM). Right: distribution of individual participant difference scores (average unisensory distractor RT – average multisensory distractor RT). (C) Left: group average participant response times to unisensory targets paired with unisensory versus multisensory low-value distractors (error bars are SEM). Right: distribution of individual participant difference scores (average unisensory distractor RT – average multisensory distractor RT). (D) Left: group average participant response times to multisensory targets paired with unisensory versus multisensory low-value distractors (error bars are SEM). Right: distribution of individual participant difference scores (average unisensory distractor RT – average multisensory distractor RT). NS = not significant. *p < .05. **p < .01. ***p < .001.

For low-value distractors, we found no difference between response times for multisensory versus unisensory distractors when the target was unisensory as predicted (unisensory distractor: *M* = 327 ms, *SD* = 37 ms; multisensory distractor: *M* = 324 ms, *SD* = 40 ms; *t*[49] = 1.49, *p =* .287), but, contrary to predictions, responses were faster to multisensory targets paired with unisensory distractors (compared to multisensory distractors; unisensory distractor: *M* = 309 ms, *SD* = 42 ms; multisensory distractor: *M* = 316 ms, *SD* = 44 ms; *t*[49] = 2.79, *p =* .03, *d =* 0.40).

## Discussion

Previously rewarded stimuli can influence later performance even when they are task-irrelevant, and multisensory signals can speed responses to task-relevant targets. Here, we examined how reward history and multisensory stimulus configuration interact in a speeded localisation task. We extended a typical VMAC-related paradigm by independently varying whether targets and distractors were unisensory visual or multisensory audiovisual stimuli. This allowed us to test whether previously rewarded distractors produced different levels of behavioural interference depending on the sensory composition of the target and distractor.

### Multisensory Target Benefit and Value-Dependent Interference

Consistent with predictions, participants responded faster to multisensory than unisensory targets, indicating a multisensory target benefit. When the target was multisensory, visual information combined with spatially and temporally congruent auditory information, increasing target processing and leading to faster attainment of the neural threshold required to initiate a response (Gingras et al., 2009; Rowland & Stein, 2008; Stanford & Stein, 2007; Stanford et al., 2005; Tang et al., 2016).

Also consistent with predictions, there was value-dependent slowing when both the target and distractor were unisensory: response times were slower for high- than low-reward distractors. This pattern is consistent with previous VMAC findings showing that previously rewarded stimuli can continue to influence behaviour even when task-irrelevant and no longer rewarded (Anderson et al., 2011b; Le Pelley et al., 2015). However, because the present task mapped the target location directly onto the response, this slowing should be interpreted as value-dependent behavioural interference rather than as uniquely indexing attentional capture.

### Multisensory Signals at the Target Reduced Value-Dependent Interference

As predicted, multisensory signals at the target interacted with distractor reward history. With multisensory targets paired with unisensory distractors, distractor value did not influence target response times. Thus, the value-dependent slowing observed when both target and distractor were unisensory was no longer evident when the target was multisensory. This effect was not simply due to the general response-time advantage for multisensory targets: although responses were faster to multisensory than unisensory targets paired with unisensory distractors, the multisensory benefit was greater when the distractor carried a high rather than a low-reward history. This pattern suggests that multisensory signals aligned with the current task can reduce the behavioural impact of previously rewarded distractors. The present data do not determine whether this reduction occurs through attentional selection, response selection, or an interaction between these processes.

These findings remain compatible with contemporary priority-map accounts in which reward history contributes to stimulus priority alongside endogenous goals and exogenous salience (Awh et al., 2012; Pearson et al., 2016; Theeuwes, 2019). However, the present localisation task does not allow us to isolate priority-map effects from response-selection effects. We therefore interpret the current findings more conservatively as showing that multisensory target signals can alter the behavioural expression of reward history in a target-localisation task.

### Multisensory Signals at the Distractor Modulated Value-Dependent Interference

Consistent with predictions, with high-value distractors, responses to targets were slower with multisensory compared with unisensory distractors, but only when the target was also multisensory. This aligns with Bean et al. (2021), suggesting that high-reward visual distractors may have been more effectively integrated with auditory tones. In the present task, this produced greater behavioural interference. This interference may reflect increased distractor processing, increased response competition, or both.

Contrary to expectations, when the target was unisensory, the predicted increase in interference from multisensory high-value distractors did not occur. Instead, response times were faster for multisensory than unisensory high-reward distractors. A related possibility comes from the “pip-and-pop” literature (Van der Burg et al., 2008), whereby a simultaneous but spatially incongruent auditory tone increases the salience of a visual target in cluttered displays, improving search efficiency (Koelewijn et al., 2010; Van der Burg et al., 2008, 2010). In our task, the auditory tone occurred simultaneously with the visual display, but, in this condition, was spatially aligned with the distractor rather than the target. Thus, rather than enhancing the target directly, the tone may have increased target–distractor distinctiveness or reduced response competition by marking the distractor as different from the unisensory target.

For low-value distractors, we found no difference between multisensory and unisensory distractors when the target was unisensory. This is broadly consistent with the possibility that low-value visual distractors were less effectively integrated with auditory tones (Bean et al., 2021; Bruns et al., 2014), although the present behavioural data cannot directly establish the strength of audiovisual integration. However, when the target was multisensory, low-value multisensory distractors impaired responses more than low-value unisensory distractors, again, contrary to predictions. One possible explanation comes from Jensen et al. (2019), who found that multisensory distractors exerted their strongest influence when overt attention was directed toward them. In our study, participants had to monitor both potential stimulus locations, potentially facilitating distractor–tone integration regardless of reward history. However, given the spatial response mapping, this effect may also have been expressed through response-level competition.

The pattern of distractor-modality effects also suggests a role for target–distractor sensory similarity in shaping target–distractor competition. Responses were generally slower when target and distractor shared the same sensory format and faster when they differed, consistent with similarity-based competition accounts. However, this factor alone cannot explain the present findings. The target and distractor were always visually distinguishable by colour, because the target was grey, whereas the distractors were blue or green, and modality configurations were unpredictable across trials, limiting the extent to which the auditory component could act as a reliable disambiguation cue. More importantly, similarity-based predictions failed in the low-value conditions, where unisensory and multisensory distractors produced equivalent performance when the target was unisensory. By contrast, reward history appeared to modulate these similarity effects, increasing interference when multisensory distractors competed strongly with multisensory targets and reducing interference when multisensory distractors were more easily distinguished from unisensory targets.

A limitation of the present design is that it cannot fully distinguish effects on attentional selection from effects on response selection. During the association phase, participants learned to make spatially defined responses to reward-associated coloured stimuli. During the test phase, those same stimuli appeared as distractors in the opposite hemifield to the target, while the required response was again defined by target location. Thus, value-dependent slowing could reflect attentional prioritisation of the previously rewarded distractor, activation of a learned distractor-associated response tendency, or an interaction between these processes. Importantly, this limitation does not undermine the central finding that reward history and multisensory stimulus configuration interact in shaping target-localisation performance. Rather, it constrains the level at which this interaction can be interpreted. We therefore frame the present findings as evidence that reward history and multisensory signals jointly modulate behavioural performance, without claiming that the effects arise uniquely at the level of attentional selection.

Taken together, our findings show that reward history and multisensory stimulus configuration interact in complex ways during target localisation. When the target was multisensory and the distractor was unisensory, value-dependent slowing was no longer evident. Conversely, when the distractor was multisensory, its effect depended on the sensory composition of the target and the prior reward value of the distractor. These results are broadly compatible with biased-competition accounts in which behaviour reflects the combined influence of current goals, stimulus salience, and selection history (Awh et al., 2012; Le Pelley et al., 2024; Theeuwes, 2019). However, because the present task confounded target location with response, the current data cannot specify whether these influences operated at the level of attentional priority, response selection, or both. The core contribution of the present study is therefore to show that reward history and multisensory signals jointly shape behavioural interference in a localisation task.

The relationship between reward history and multisensory stimulus configuration remains less clear when both distractor and target are multisensory. In our data, multisensory distractors at all reward levels were more distracting than their unisensory counterparts when the target was multisensory. It is difficult to determine whether this reflects a general increase in competition from multiple multisensory stimuli, a reward-related modulation of distractor processing, response-level conflict, or some combination of these factors. Recent VMAC work likewise suggests that the behavioural expression and persistence of value-related interference can depend on task context, explicit reward knowledge, and awareness of reward contingencies (Garre-Frutos, Ariza, & González, 2025; Garre-Frutos, Lupiáñez, & Vadillo, 2025). The present findings extend this broader point by showing that multisensory stimulus configuration also shapes how reward history is expressed behaviourally. Following on the contribution of MSI, future studies could address this by using previously rewarded stimuli as both targets and distractors in the test phase, systematically pairing high- and low-reward items across unisensory and multisensory conditions to directly compare value-dependent behavioural interference across conditions.

Future studies could more directly separate attentional and response-selection accounts by making the required response orthogonal to target and distractor location, for example by requiring participants to respond to a non-spatial target feature while target and distractor location vary independently.

Beyond their theoretical contribution, these findings have potential applied relevance. Understanding how reward history and MSI interact could inform the design of more effective performance-support strategies in contexts such as driving assistance systems (Steenken et al., 2014) or interventions for addiction-related disorders like gambling and alcohol dependence (Anderson et al., 2011b). More broadly, pairing task-relevant but less intrinsically rewarding information with multisensory cues may offer a practical means of biasing processing toward goal-aligned options, potentially supporting faster and more accurate decision-making in situations where timing is critical.

## Data Availability

The data, materials, and analysis scripts for this study are publicly available on the Open Science Framework (OSF) at https://osf.io/akdx7/files/osfstorage/6932348d8318814408ca08f4. The study was not preregistered.

## Notes

1. Similar findings from other motivational learning contexts support the notion that MSI is sensitive to top-down factors including emotional valence (Maiworm et al., 2012) and feedback training (Rosenthal et al., 2009).
